# Socioeconomic determinants of accessibility to birth registration in Lao PDR

**DOI:** 10.1186/s12889-017-5009-x

**Published:** 2018-01-08

**Authors:** Marika Nomura, Phonepadith Xangsayarath, Kenzo Takahashi, Yusuke Kamiya, Latsamy Siengsounthone, Hina Ogino, Jun Kobayashi

**Affiliations:** 1 Department of International Health and Collaboration, National Institute of Public Health, 2-3-6 Minami, Wako-shi, Saitama, 351-0197 Japan; 2 National Institute of Public Health, Vientiane, Lao PDR; 3 Teikyo University, Tokyo, Japan; 4 Ryukoku University, Kyoto, Japan; 5 Yokkaichi Nursing and Medical Care University, Mie, Japan; 6 Ryukyu University, Okinawa, Japan

**Keywords:** Birth registration, Socioeconomic factors, Capacity development, Home delivery, Lao PDR

## Abstract

**Background:**

The global coverage rate of birth registration is only around 65% for the population of children under five although birth registration secures protection and access to health services that are fundamental rights for all babies. This study aimed to perform a basic analysis of the accessibility to birth registration to better understand how to improve the birth registration system in the Lao PDR.

**Methods:**

For the analysis of birth registration and related socioeconomic factors, 9576 mother-child pairs were chosen from the data set of The Lao Social Indicator Survey 2011–12. After bivariate analysis with statistical tests including the chi-square test were conducted, logistic regression was performed to determine the variables that statistically influence accessibility to birth registration.

**Results:**

Ethno-geographic factors and place of delivery were observed to be the factors associated with birth registration in this analysis.

**Conclusion:**

Many mothers in the Lao PDR deliver in their local communities. Therefore, capacity development of various human resources, such as Skilled Birth Attendant, to support the local administrative procedure of birth registration in their communities could be one option to overcoming the bottlenecks in the birth registration process in the Lao PDR.

## Background

Under the Convention of the Rights of the Child, all children have the right to have a legally registered name, to be officially recognized by the government and also to have a certified nationality [1]. However, globally, almost 230 million children have never been officially registered according to a 2013 UNICEF report [2]. Birth registration secures protection and access to health services that are fundamental rights for all babies. According to previous reports, political stance, law, human rights and economic, cultural, gender, geographical and domestic security issues are cited as possible bottlenecks in birth registration [3, 4].

Basic demographic data collected by birth registration are crucial information for national planning and monitoring because they enable the creation of effective strategies not only for the health sector but for all sectors of development [5, 6]. The registration of births is fundamental to ensure civil and political rights including those of enrollment of children in school at the appropriate age and their right of access to appropriate healthcare [7]. In addition, the legal acknowledgement of a child’s existence protects the child from various deprivations such as child trafficking, under-age participation in the military and forced marriage [5, 8].

According to the 2013 UNICEF report, the coverage rate of birth registration is only around 65% of the population of children under five worldwide [2]. The low coverage of birth registration in Asian and Sub-Saharan African countries implicates various complicated factors such as geographical conditions or socioeconomic situations as possible bottlenecks, especially in remote communities. The results from Ghana indicated that birth registration is a privilege for children whose parents are educated, wealthy and living in urban communities [9]. In Nigeria, age, level of education, marital status, occupation, place of delivery, attended antenatal care and food availability at home were the factors related to the accessibility to birth registration [10, 11]. Although the bottlenecks have been investigated from various angles in Africa, exploration of the socioeconomic factors of the practice of birth registration in Southeast Asia remains inadequate.

The Lao PDR, the subject country of this analysis, has a rate of birth registration of 75% according to the 2015 UNICEF report [12]. This figure, along with other maternal and child health indicators, is one of the lowest among the ASEAN countries (Table 1). In the Lao PDR, a series of family registrations, such as birth registration, is provided for under the “Family Registration Law” [13]; however, the actual practice of birth registration in the community seems to be hard to achieve because in the Lao PDR, many mothers deliver their children at home in their local communities, which accounts for 75% of the total deliveries. In this unique context, delivery conditions, which are included as a part of social determinants [14], are likely to be a predominant factor related to the accessibility to birth registration in the Lao PDR [12].

**Table 1:** Indicators of maternal, newborn and child health in the ASEAN countries

Countries	Infant mortality rate (under 1 year)	Neonatal mortality rate	Maternal mortality ratio (Adjusted)	Maternal mortality ratio (Reported)	Skilled attendant at birth	Institutional delivery	Antenatal care at least one visit	Antenatal care at least four visit	Birth registration, 2005–2013
Indonesia	25	14	190	360	83	63	96	88	67
Philippines	24	14	120	220	72	55	95	78	90
Vietnam	19	13	49	67	93	92	94	60	95
Thailand	11	8	26	12	100	100	98	93	99
Myanmar	40	26	200	320	71	36	83	73	72
Malaysia	7	4	29	26	99	99	97	–	–
Cambodia	33	18	170	210	72	61	89	59	62
Lao PDR	54	29	220	360	42	38	54	37	75
Singapore	2	1	6	–	–	100	–	–	–
Brunei	8	5	27	–	100	100	99	–	–
Southeast Asia average	61	40	74	–	93	87	94	80	79

Source: The State of the World’s Children 2015

Therefore, this study aimed to perform a basic analysis of the accessibility to birth registration to better understand methods to improve the birth registration system in the Lao PDR and to determine policy implications for overcoming the difficult achievement of birth registration in Lao PDR, where most mothers choose home delivery.

## Methods

### Data

We used data from the 2011–12 Lao Social Indicator Survey (LSIS) in the present analysis. The LSIS is a household-based survey that applies the technical frameworks of the Multiple Indicator Cluster Survey and the Demographic and Health Survey [15]. LSIS was conducted to monitor the progress towards the Millennium Development Goals and to serve as a baseline for the 7th National Socio-Economic Development Plan [16]. Field data were collected from October 2011 to February 2012. Among the 18,843 households interviewed nationally in the survey, 97,421 household members were listed. Of these, 47,820 were men and 49,601 were women. The average household size found in the survey was 5.2 persons.

The response rates for households, women and men were 99% (18,843/19,018), 94% (22,476/23,937) and 89% (9951/11,166), respectively. Detailed information was obtained via interview on 11,067 (98%) of the 11,258 children under 5 years of age listed in the household questionnaire. Face-to-face interviews were conducted with all women aged 15–49 years and men aged 15–59 years in the sampled households by use of questionnaires covering socioeconomic, demographic and health indicators.

The survey covers a broad range of topics including water and sanitation, marriage and sexual activity, fertility levels and trends, reproductive health, adult and maternal mortality, child health, nutrition, child mortality, child development, literacy and education, child protection, HIV/AIDS and sexual behavior, access to mass media and use of information/communication technology.

### Statistical analysis

Values related to the accessibility to birth registration across the categories of the explanatory variables are presented as numbers and percentages. The outcome indicator of birth registration includes children whose parents self-reported the possession of a birth certificate and children whose mother or caretaker said that the birth had been registered [15].

From the LSIS data set, 9576 mother-child pairs for which complete data on socioeconomic stat was available were used for the analysis of birth registration and related socioeconomic factors.

First, bivariate analysis was conducted to explore the proportion of children registered with respect to each socioeconomic factor. Statistical tests including the chi-square test were used to test whether there were significant associations between the responses about birth registration and the socioeconomic factors. The analysis used 14 socioeconomic factors at the child, maternal, household and community levels: child’s sex, child’s age in months, maternal age, marital status, maternal educational attainment, experience of delivery, experience of child loss, paternal educational attainment, head of household’s ethnicity, head of household’s ethno-language, head of household’s religion, wealth index quintiles, region and settlement. Bivariate analysis was also performed to clarify the relationships between place of delivery, delivery attendance and birth registration. Finally, we used logistic regression to explore the variables that statistically influenced accessibility to birth registration by preparing 3 logistic models (model 1 included “maternal, household and community variables only”; model 2 included “maternal, household, community variables and skilled birth attendant (SBA) attendance”; and model 3 included “maternal, household, community variables, SBA attendance and place of delivery”). All analyses were done using IBM SPSS Statistics for Windows, Version 24.0.

## Results

**Fig. 1:**
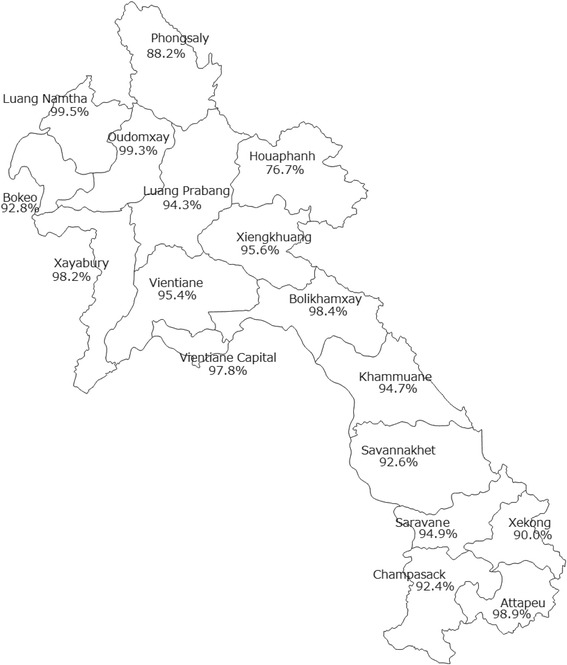
Geographical distribution of the percentages of birth registrations by province in the Lao PDR. The original map was retrieved from the URL below and licensed under the Creative Commons Attribution 2.5 Generic license. Https://commons.m.wikimedia.org/wiki/File:Laos_provinces_blank.png is provided by Wikipedia with common license

Figure 1 shows the geographical distribution of birth registration among the 17 provinces in the Lao PDR. Apart from most of the provinces, which have almost 100% birth registration, only Huaphanh province in the North had a significantly low percentage of 76.7%. Vientiane Capital, site of the national capital, reported relatively high coverage of birth registration.

Table 2 presents the proportions of children between the ages 0–59 months who have birth registration according to child, maternal, household and community factors. Corresponding levels of association between the independent variables and the dependent variable are also indicated based on chi square test results. There was no association between child sex and birth registration. Children whose mothers had experienced child loss had less access to birth registration. Children whose parents had higher levels of education were more likely to have birth registration than those whose parents had a lower level of education. For instance, rates of access to birth registration by children according to the mother’s educational level were as follows: no education, 66.3%; primary education, 75.1%; secondary education, 83.9% and higher education, 93.8%. The trend was clearer than that for the father’s educational attainment. Rates of access to birth registration by children according to the father’s educational level were as follows: no education, 66.6%; primary education, 71.1%; secondary education, 79.6% and higher education, 92.7%. Similar patterns were observed with higher household wealth. In terms of ethnicity, only 57.1% of the Khmer ethnic group had access to birth registration. Only 68.1% of Animists had access to birth registration, in contrast to 81.4% of Buddhists.

**Table 2:** Relationship between child factors, maternal factors, household factors, community factors and birth registrations, Lao PDR (*n* = 9576)

		Birth registration				
Independent factors		Yes		No		P^a^
		n	(%)	n	(%)	
Child factors						
Child sex						
	Male	3580	73.7	1276	26.3	0.174
	Female	3537	74.9	1183	25.1	
Child age^b^ (months)						
	0–11	1222	60.4	796	39.4	<0.001
	12–23	1406	74.0	494	26.0	
	24–35	1410	76.4	436	23.6	
	36–47	1589	80.7	379	19.3	
	48–59	1490	80.8	354	19.2	
Maternal factors						
Maternal age^b^ (years)						
	15–19	443	65.4	234	34.6	<0.001
	20–29	4041	74.5	1386	25.5	
	30–39	2179	76.1	686	23.9	
	40–49	454	74.8	153	25.2	
Marital status						
	Never	30	71.4	12	28.6	0.667
	Married	7083	74.3	2447	25.7	
	Divorced	2	100.0	0	0.0	
	Widowed	2	100.0	0	0.0	
Maternal educational attainment^b^						
	No education	2229	66.3	1131	33.7	<0.001
	Primary	2969	75.1	982	24.9	
	Secondary	1738	83.9	334	16.1	
	Higher	181	93.8	12	6.2	
Ever given birth						
	Yes	7105	74.3	2458	25.7	0.137
	No	12	92.3	1	7.7	
Ever experienced loss of a child^b^						
	Yes	1703	69.2	759	30.8	<0.001
	No	5414	76.1	1700	23.9	
Household factors						
Father’s educational attainment^b^						
	No education	1015	66.6	510	33.4	<0.001
	Primary	3094	71.1	1257	28.9	
	Secondary	2575	79.6	658	20.4	
	Higher	433	92.7	34	7.3	
Ethnicity of HHH^b^						
	Lao	2911	83.2	588	16.8	<0.001
	Khmu	1056	79.8	268	20.2	
	Khmer	764	57.1	575	42.9	
	Other	2386	69.9	1028	30.1	
Ethno-linguistics of HHH^b^						
	Lao-Thai	3472	81.1	808	18.9	<0.001
	Mon-Khmer	2482	73.9	878	26.1	
	Hmong-Mien	805	57.6	593	42.4	
	Chinese-Tibetan	358	66.5	180	33.5	
Religion of HHH^b^						
	Buddhist	3576	81.4	815	18.6	<0.001
	Animist	3447	68.1	1616	31.9	
	Other	94	77.0	28	23.0	
Wealth index quintiles^b^						
	Poorest	2186	66.9	1079	33.1	<0.001
	2nd quintile	1546	69.4	683	30.6	
	3rd quintile	1336	77.7	384	22.3	
	4th quintile	1077	81.7	241	18.3	
	Wealthiest	972	93.1	72	6.9	
Community factors						
Region^b^						
	North	2558	67.2	1249	32.8	<0.001
	Central	2569	80.4	627	19.6	
	South	1990	77.3	583	22.7	
Settlement^b^						
	Urban	1528	88.4	200	11.6	<0.001
	Rural w/ road	4926	72.8	1836	27.2	
	Rural w/o road	663	61.0	423	39.0	
Total		7117	74.3	2459	25.7	

HHH: head of household

^a^*P*-value was calculated for chi square tests (categorical variables)

^b^Correlation between the independent variable and birth registration/birth certificate is statistically significant at the 0.05 level, and the variable was included in further logistic regression analyses

In regard to community factors, there were broad-based variations in birth registration by region as shown in Fig. 1. In addition, settlement was likely to be related to the rates of attainment of birth registration: 88.4% in urban areas versus 61.0% in rural areas without roads.

The results of accessibility to birth registration by delivery condition were significantly different (Table 3). Children who were born at health centers (68.4%) and at home (65.0%) were less likely to have their birth registered compared to those born at a hospital (85.1%) or a private facility (89.5%). Attendance by a SBA seem to encourage mothers to obtain birth registration. In contrast, delivery with a traditional birth attendant/community health worker (TBA/CHW) did not seem to result in mothers obtaining a birth registration for their newborns; there was no significant difference between TBA/CHW and birth registration.

**Table 3:** Relationship between delivery place and birth attendance and birth registration, Lao PDR

		Birth registration				
Independent factors		Yes		No		P^a^
		n	(%)	n	(%)	
Delivery place^b^						
	Hospital	1240	85.1	217	14.9	<0.001
	Health center	130	68.4	60	31.6	
	Private facility	34	89.5	4	10.5	
	Home	2535	65.0	1368	35.0	
	Other	73	38.4	117	61.6	
Skilled birth attendant^b^						
	Yes	1422	79.3	370	20.7	<0.001
	No	5695	73.2	2089	26.8	
TBA/CHW						
	Yes	588	72.2	226	27.8	0.155
	No	6529	74.5	2233	25.5	

TBA/CHW: Traditional birth attendant/Community health worker

^a^P-value was calculated for chi square tests (categorical variables)

^b^Correlation between the independent variable and birth registration/birth certificate is statistically significant at the 0.05 level, and the variable was included in further logistic regression analyses

Logistic regression analysis was applied to determine the variables that statistically influenced the attainment of birth registration. Variables that were of no significance, variables related to the child and variables that had collinearity were excluded from the analysis beforehand. The results are presented in Table 4.

**Table 4:** Odds ratios (95% CIs) of birth registration by different models, Lao PDR

		Birth registration								
		*Model 1* ^*a*^			*Model 2* ^*b*^			*Model 3* ^*c*^		
		*OR*	*95% CI*		*OR*	*95% CI*		*OR*	*95% CI*	
Maternal factors										
Maternal age										
	15–19	1.00			1.00			1.00		
	20–29	1.45	1.21	1.73***	1.45	1.21	1.73***	1.35	1.12	1.64***
	30–39	1.67	1.37	2.02***	1.67	1.37	2.02***	1.54	1.24	1.91***
	40–49	1.83	1.41	2.37***	1.83	1.41	2.37***	1.49	1.06	2.10**
Maternal educational attainment										
	No education									
	Primary									
	Secondary									
	Higher									
Ever experienced loss of a child										
	No	1.00			1.00					
	Yes	0.86	0.76	0.96**	0.86	0.76	0.96**			
Household factors										
Father’s educational attainment										
	No education	1.00			1.00			1.00		
	Primary	1.05	0.91	1.19	1.05	0.91	1.19	0.96	0.81	1.13
	Secondary	1.19	1.02	1.40 *	1.19	1.02	1.40 **	1.11	0.92	1.35
	Higher	2.04	1.37	3.05***	2.04	1.37	3.05***	2.03	1.30	3.18***
Ethnicity of HHH										
	Lao	1.00			1.00			1.00		
	Khmu	1.08	0.82	1.41	1.08	0.82	1.41	0.86	0.62	1.18
	Khmer	0.37	0.20	0.68***	0.37	0.20	0.68***	0.42	0.19	0.92**
	Other	0.63	0.52	0.77***	0.63	0.52	0.77***	0.59	0.47	0.75***
Ethno-linguistics of HHH										
	Lao-Thai	1.00			1.00			1.00		
	Mon-Khmer	1.40	1.14	1.72***	1.40	1.14	1.72***	1.64	1.27	2.12***
	Hmong-Mien	1.20	0.67	2.18	1.20	0.67	2.18	1.12	0.51	2.44
	Chinese-Tibetan	1.20	0.93	1.56 **	1.20	0.93	1.56	1.07	0.77	1.47
Religion of HHH										
	Buddhist	1.00			1.00					
	Animist	0.89	0.75	1.05	0.89	0.75	1.05			
	Other	1.51	0.96	2.40**	1.51	0.96	2.40*			
Wealth index quintiles										
	Poorest	1.00			1.00					
	2nd quintile	1.00	0.88	1.13	1.00	0.88	1.13			
	3rd quintile	1.34	1.15	1.57***	1.34	1.15	1.57***			
	4th quintile	1.40	1.16	1.70***	1.40	1.16	1.70***			
	Wealthiest	2.78	2.05	3.77***	2.78	2.05	3.77***			
Community factors										
Settlement										
	Urban	1.00			1.00			1.00		
	Rural w/ road	0.62	0.52	0.74 ***	0.62	0.52	0.74***	0.63	0.51	0.77***
	Rural w/o road	0.39	0.31	0.49***	0.39	0.31	0.49***	0.38	0.30	0.49***
Delivery factors										
Delivery place										
	Hospital							1.00		
	Health center							0.53	0.38	0.76***
	Private facility							1.56	0.54	4.55
	Home							0.47	0.39	0.56***
	Other							0.15	0.11	0.22***
SBA assisted										
	No									
	Yes									
N		9576			9576			5778		
Log likelihood		10,127.238			10,127.238			6589.391		
*X*^2^ test for model		<0.01			<0.01			<0.01		
Hosmer-Lemeshow		0.369			0.369			0.233		

CI: confidence interval; HHH: head of household; OR: odds ratio; SBA: skilled birth attendant

*p < .10, ** *p* < .05, *** *p* < .01

^a^Model 1: maternal, household and community variables only

^b^Model 2: maternal, household, community variables and birth attendance

^c^Model 3: maternal, household, community variables, birth attendance and delivery place

Maternal age was the significant factor for birth registration, rather than maternal educational attainment, in model 1. Higher educational attainment of the father and higher household wealth were likely to accelerate registration of a child’s birth. Khmer people were significantly less likely to have access to birth registration. SBA attendance was not associated with accessibility to birth registration. SBA attendance was excluded in model 2 and thus had no association with birth registration. In model 3, place of delivery, experience of child loss, religion and household index were not associated with birth registration in contrast to the results in models 1 and 2. As expected, children who were born in a community were less likely to have birth registration: 46% in health centers, 53% at home and 85% in other locations.

## Discussion

In this analysis, we illustrated the geographical distribution of birth registration, and then we examined variables related to the outcomes of birth registration. Ethnicity of the head of the household, settlement and delivery place were observed to be the important factors related to accessibility of birth registration in this analysis.

First, considerable geographical distribution was identified across all of the provinces. Accessibility in Huaphanh province and Pongsaly province in the Northern area was quite low compared with that in the Central and Southern areas. In the Lao PDR, the Northern area, in which ethnic minorities dwell, consists of a mountain range running along the border with China, Myanmar and Vietnam. In the multivariable analysis, settlement and ethnicity were the common factors that remained in all models, which means that ethno-geographic factors may determine the accessibility to birth registration. Even after adjustment for SBA attendance or place of delivery, settlement and ethnicity were still likely to be significant factors. Of great concern are the disparities between different regions in the nation, within regions and between urban and rural areas [2]. The possible reasons accounting for these ethno-geographic differences in birth registration in the Lao PDR are poor literacy in ethnic minority groups and poor accessibility by road especially in the Northern mountainous region. Thus, we surmise that the birth registration system is not working well in the mountainous remote areas where ethnic minorities dwell. For this reason, a community-based strategy targeting the people living in remote areas or ethnic minority groups is needed.

Because geographical disparity is a fundamental issue that cannot be avoided, we should further promote community-based intervention, which is in place everywhere in the Lao PDR. Considering the geographical disparities of accessibility to birth registration in many communities, community health volunteers (CHVs) assume a key role in issuing birth certificates and supervising the management of the family book in the Lao PDR. In some regions, CHVs work actively and have all of the information on vital statistics such as births or deaths [17–19]. Strengthening the monitoring and evaluation system of vital statistics by further enhancing the capacity of CHVs would be a useful measure because they may play an important role as the recorder of vital statistics in remote communities. Empowering village heads and administrative staffs who are in charge of managing vital statistics in a village to monitor the community population could be another possible approach. Furthermore, from a gender perspective, the father’s higher education attainment was significantly associated with birth registration. Approaches to enlighten fathers’ awareness and knowledge about birth registration may exert a rapid effect on increasing the rate of birth registration in the Lao PDR. Such efforts will serve as a fundamental service from the government in the future.

In the multivariate analysis, not surprisingly, place of delivery was significantly associated with birth registration: the children who were born at a health center, at home or at other places were especially much less likely to have accessibility to birth registration. Only 65.0% of the children who were born at home had access to birth registration. This is 10 percentage points lower than the national representative average rate of birth registration of 75% [12]. Widely throughout Southeast Asian countries, and especially in the Lao PDR, home is the major place for the delivery of children [20, 21]. One of the reasons for choosing home delivery is that Lao mothers expect to follow the traditional habit of staying by the fire during the perinatal period [20].

To improve access to proper birth registration especially in pockets of the Lao PDR, one possible approach could be capacity building of SBAs in the community to support mothers’ access birth registration right after delivery [22]. The WHO Western Pacific Region Office has set a target to increase SBA-assisted delivery coverage to 90% by 2020 [23]. SBAs should instruct parents who choose a community-based delivery in the birth registration procedure. Although not addressed in this paper, 29.3% of children obtained a birth certificate. To guarantee continuum of care, improvement of the procedures to obtain not only birth registration but also to acquire a birth certificate are important issues. For maternal and child health, it is necessary to strengthen antenatal care and safe motherhood, the continuum of care leading to parental care, and the life-cycle approach throughout life. Birth certificates may be necessary for various events in life, such as for education or overseas travel. SBAs should also be required to play a role in assisting in the acquisition of birth certificates. For the Lao PDR, visualizing and counting all people, including those who are marginalized, is the most crucial step also from the perspective of universal health coverage (UHC) [24].

Although this study reached its aims and important policy implications can be drawn from the results, it does have an important limitation. The study was carefully prepared by conducting a statistical analysis using representative national data, but it is a secondary analysis using national data with limited available variables. Thus, conclusions about causality cannot be drawn, nor can all possible confounders be taken into account. Therefore, in terms of UHC, our analysis raises further questions about what are the other factors related to achieving birth registration in rural communities in the Lao PDR. Further investigation of birth registration is recommended to collect detailed community-based data that can contribute to improving accessibility to the birth registration system in the Lao PDR.

## Conclusions

Our findings confirmed that ethno-geographic factors and place of delivery appear to be the two most significant factors associated with accessibility to birth registration in the Lao PDR. As one effective strategy to overcoming geographical disparity in the Lao PDR, where most mothers choose home delivery, we recommend community-based interventions that focus on capacity development of community health personnel such as the SBA.

## Abbreviations

ASEAN: Association of Southeast Asian Nations
CHV: community health volunteer
CHW: community health worker
Lao PDR: Lao People’s Democratic Republic
LSIS: Lao Social Indicator Survey
SBA: skilled birth attendant
SPSS: Statistical Package for Social Sciences
TBA: traditional birth attendant
UHC: universal health coverage
UNICEF: United Nations International Children’s Emergency Fund
WHO: World Health Organization
